# Dentists’ Knowledge and Clinical Preferences Regarding Evidence-Based Restorative Material Selection in Türkiye: Implications for Clinical Practice and Healthcare Policy

**DOI:** 10.3390/healthcare14091129

**Published:** 2026-04-23

**Authors:** Mehmet Salık, Elif Pınar Bakır, Şeyhmus Bakır

**Affiliations:** 1 Department of Restorative Dentistry, Faculty of Dentistry, Dicle University, Diyarbakır 21280, Türkiye; elifpinarbakir@gmail.com; 2 Department of Restorative Dentistry, Faculty of Dentistry, Batman University, Batman 72000, Türkiye; seyhmusbakir@gmail.com

**Keywords:** evidence-based dentistry, restorative material selection, clinical preferences, evidence–practice gap

## Abstract

**Background/Objectives:** This study aimed to evaluate the self-reported knowledge levels of evidence-based practice among actively practicing dentists in Türkiye in relation to restorative material selection, and to identify the demographic and professional factors associated with this knowledge. **Materials and Methods:** This cross-sectional descriptive survey included 341 dentists and was conducted using an online questionnaire. Descriptive statistics were expressed as frequencies, percentages, means, and standard deviations. Factors associated with self-reported knowledge levels of evidence-based dentistry were initially assessed using ordinal logistic regression. As the proportional odds assumption was violated, multinomial logistic regression analysis was employed as the final model. Statistical significance was set at *p* < 0.05. **Results:** Aesthetic expectations and durability were identified as the primary factors influencing restorative material selection. Approximately half of the participants reported low-to-moderate levels of knowledge regarding evidence-based dentistry. Multinomial logistic regression analysis demonstrated that professional experience, professional status, and type of institution were significant predictors of knowledge level (*p* < 0.05). General dental practitioners were significantly less likely to have high knowledge levels compared to specialists. Certain experience groups also showed a lower likelihood of achieving higher knowledge levels relative to the reference group. Although the type of institution was statistically significant, its overall effect was limited. **Conclusions:** The findings indicate that knowledge of evidence-based approaches in restorative material selection among dentists in Türkiye remains suboptimal. While professional status and experience play a significant role in shaping knowledge levels, institutional factors appear to have a comparatively minor impact. These results underscore the need for structured continuing education programmes—particularly for general dental practitioners—and highlight the importance of strengthening evidence-based decision-making in clinical practice.

## 1. Introduction

Decision-making in restorative dentistry, particularly in the selection of restorative materials, is a complex and multifactorial process influenced by a wide range of clinical, material-related, and patient-specific factors [1]. From the clinician’s perspective, material selection is primarily influenced by patient-specific characteristics, the clinician’s level of education and clinical experience, as well as the type of restoration being performed [2,3]. At the patient level, factors such as caries risk, parafunctional habits (e.g., bruxism), the size of the planned restoration, the presence of endodontic treatment, and the intraoral position of the tooth have been shown to play a determining role in this process [4].

In restorative material selection, the application of evidence-based dentistry is expected to guide clinical decision-making. However, clear and consistent evidence is not available for many materials [5]. Although this approach aims to ensure that clinical decisions are guided by reliable scientific evidence, the literature indicates that a substantial gap persists between evidence and clinical practice. This discrepancy, often referred to as the “knowledge-to-practice gap,” may lead to unnecessary interventions, suboptimal treatment outcomes, and an increased burden on healthcare systems [6,7,8].

The rapid evolution of restorative materials and the continuous introduction of new products to the market make it increasingly difficult for dentists to keep up with current developments. In addition, clinicians may not always have adequate access to up-to-date evidence-based information. As a result, material selection is often influenced by information obtained through industry representatives or professional training activities, which may be supported by limited levels of scientific evidence [9]. Experienced dentists may develop individualized approaches, as they have greater opportunities to observe the clinical performance of restorative materials [10]. In contrast, newly graduated dentists may tend to rely on the materials and techniques acquired during their undergraduate education.

The challenging conditions of the oral cavity—such as bacterial activity, high occlusal forces, fluctuating pH levels, temperature variations, and a moist environment—may complicate restorative material selection [11]. In addition, the growing global interest in aesthetic dentistry has significantly influenced decision-making in clinical practice. In this context, there is a need to develop evidence-based guidelines that can be adapted to dental practice and aim to standardize treatment processes, thereby facilitating the effective translation of scientific evidence into routine care [12,13].

Although the existing literature has demonstrated that clinical decision-making processes and material selection in restorative dentistry are influenced by a wide range of factors [1,2,3,4], there is limited evidence regarding how actively practicing dentists in Türkiye make restorative material choices in their daily clinical practice and the extent to which these decisions align with the principles of evidence-based dentistry. In particular, considering recent international policy developments such as the Minamata Convention [14] and the growing preference for tooth-colored restorative materials, there is an increasing need to evaluate this issue using up-to-date data.

Therefore, the present cross-sectional survey aimed to assess dentists’ self-reported knowledge levels regarding evidence-based restorative material selection in Türkiye, to describe their material preferences across different clinical scenarios, and to identify the demographic and professional factors associated with higher levels of knowledge. The findings of this study are expected to contribute to the development of future national clinical guidelines and continuing professional education programs.

## 2. Materials and Methods

### 2.1. Study Design and Participants

This study was designed as a descriptive cross-sectional survey to evaluate dentists’ knowledge levels and preferences regarding evidence-based restorative material selection.

The study population consisted of general dental practitioners and specialist dentists actively practicing in Türkiye. Participants were recruited using a convenience sampling method. Data collection was conducted online in 2025, and the survey link was distributed via WhatsApp through professional dental groups and communication networks.

To ensure that the sample was not limited to a specific subgroup, the survey was disseminated across multiple professional communication platforms, aiming to achieve a heterogeneous participant profile. Accordingly, dentists working in public institutions, universities, and private practice settings were included, representing a range of age groups, levels of professional experience, and institutional affiliations.

Participation in the study was entirely voluntary, and no personally identifiable information was collected. Participants were informed of their right to withdraw from the study at any time without providing a reason.

### 2.2. Inclusion and Exclusion Criteria

The inclusion criteria were as follows: (i) being a general dental practitioner or specialist dentist actively practicing in Türkiye; (ii) voluntarily agreeing to participate in the online survey; and (iii) completing all mandatory items in the questionnaire.

The exclusion criteria included: (i) being a dental student or trainee; (ii) not actively practicing dentistry (e.g., retired or working outside the profession); and (iii) submitting incomplete responses or multiple entries from the same participant.

**Figure 1:**
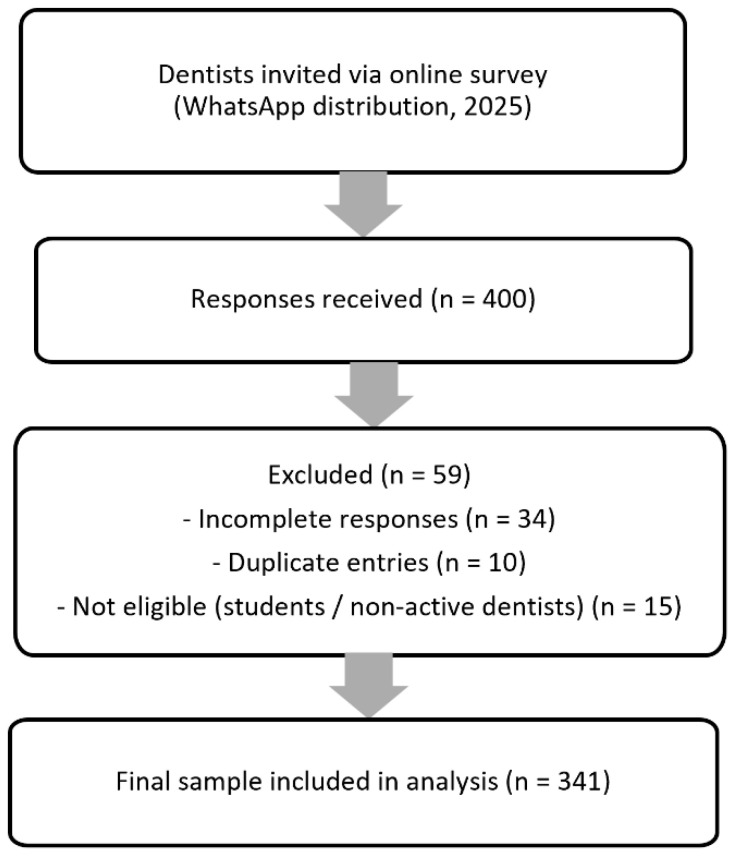
Flowchart of participant selection process.

In accordance with these criteria, a preliminary screening was conducted, and ineligible, incomplete, or duplicate responses were excluded from the final analysis (Figure 1).

### 2.3. Data Collection Instrument

Data were collected using a structured 17-item questionnaire. A pilot study was conducted prior to the main data collection to evaluate the feasibility, clarity, and technical functionality of the instrument. The pilot included 20 dentists actively practicing in Türkiye, who were subsequently excluded from the final analysis. Based on the pilot results, minor revisions were made to improve item clarity, and adjustments were applied to the question sequence and response options. The questionnaire was then considered appropriate for the main data collection.

The questionnaire was developed by the researchers based on an extensive review of the literature and current clinical guidelines [2,15]. Content validity was ensured through independent assessments by two experts in Restorative Dentistry, and items were revised in terms of clarity, relevance, and representativeness.

The instrument consisted of two main sections. The first section included questions designed to assess participants’ demographic and professional characteristics (gender, age, years of professional experience, type of institution, and professional status). The second section evaluated dentists’ awareness of evidence-based dentistry, self-reported knowledge levels, sources of information, clinical decision-making processes, and restorative material preferences across different clinical scenarios.

Participants’ self-reported knowledge levels regarding evidence-based dentistry were assessed using a 5-point Likert scale (none, low, moderate, sufficient, very good). For analytical purposes, responses were categorized into three levels: low (scores 1–2), moderate (score 3), and high (scores 4–5).

The study was conducted in accordance with the Declaration of Helsinki. Participants were provided with detailed information regarding the study procedures, confidentiality, and voluntary participation, and electronic informed consent was obtained. Ethical approval was obtained from the Ethics Committee of Dicle University (Approval No: 2025-49; Date: 25 June 2025).

### 2.4. Statistical Analysis

All statistical analyses were performed using IBM SPSS Statistics software (version 31.0; IBM Corp., Armonk, NY, USA). Descriptive statistics, including frequencies, percentages, means, and standard deviations, were used to summarize participants’ demographic characteristics and response distributions.

To determine the factors associated with self-reported knowledge levels of evidence-based dentistry, an ordinal logistic regression model was initially applied. The proportional odds assumption was assessed using the Test of Parallel Lines and was found to be violated (*p* < 0.001). Accordingly, multinomial logistic regression analysis was conducted as the final model. Statistical significance was set at *p* < 0.05.

## 3. Results

A total of 400 responses were obtained. After excluding incomplete, duplicate, and ineligible responses, 341 participants were included in the final analysis.

The majority of participants were aged between 26 and 30 years (41.9%), followed by the 31–35 and 36–40 age groups, each representing 14.7% of the sample. Participants aged ≤25 years accounted for 10.9%, while those aged ≥51 years comprised 7.6%. Overall, 67.4% of the participants were aged 35 years or younger. The mean age was 33.46 ± 8.76 years (Table 1).

Of the participants, 58.4% (*n* = 199) were male and 41.6% (*n* = 142) were female. Regarding professional experience, nearly half of the participants (49.3%) had 0–5 years of experience, followed by 15.5% with 6–10 years, 13.8% with 11–15 years, 6.7% with 16–20 years, and 14.7% with ≥21 years. In terms of professional status, the majority were general dental practitioners (68.3%), followed by research assistants (15.5%) and specialists (16.1%).

A total of 51.0% of participants were employed in public hospitals/Oral and Dental Health Centers, followed by 26.7% in universities, 19.9% in private practice, and 2.3% in other institutions.

With regard to evidence-based dentistry (EBD), 63.6% of participants reported prior awareness of the concept, whereas 36.4% had not previously heard of it. Self-reported knowledge levels were distributed as follows: 45.8% low (scores 1–2), 35.4% moderate (score 3), and 18.8% high (scores 4–5) (Table 2).

**Figure 2:**
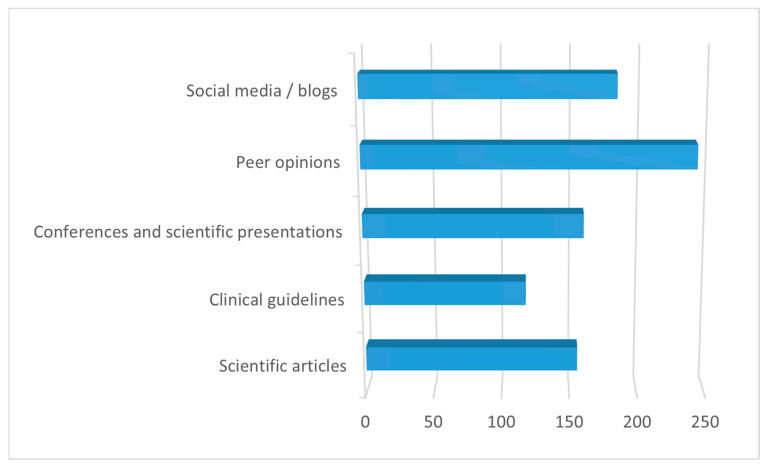
Main sources of information for post-graduation restorative material selection (participants were allowed to select multiple responses).

Among the information sources used for post-graduation restorative material selection, peer opinions were the most frequently reported (70.7%), followed by social media/blogs (53.7%), conferences and scientific presentations (46.9%), and scientific articles (45.7%). Clinical guidelines were less frequently reported, with a usage rate of 34.6% (Figure 2).

Participants reported that their knowledge of evidence-based material selection was primarily acquired through undergraduate theoretical education (59.5%) and clinical training (56.2%). Other commonly reported sources included clinical experience and peer opinions (58.0%), continuing professional courses (34.6%), scientific journal articles (33.7%), and professional meetings and congresses (32.2%). Online educational platforms and webinars were less frequently reported (12.4%) (Table 3).

When the frequency of reviewing the composition and safety information of restorative materials was assessed, 66.6% of participants reported reviewing such information when introducing a new material, 12.4% reported regularly following technical documentation, and 21.0% reported rarely or never considering this information.

With respect to biocompatibility considerations, 64.8% of participants reported evaluating biocompatibility based on scientific evidence, whereas 27.0% relied on manufacturer-provided information and 7.3% reported not considering biocompatibility.

**Figure 3:**
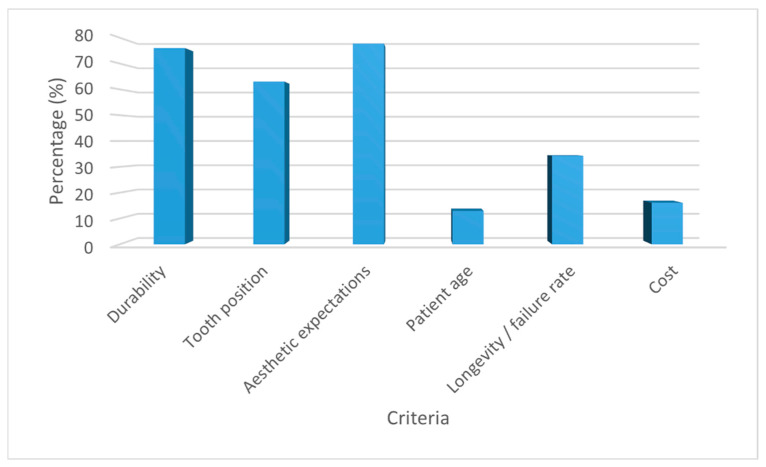
Factors influencing resin composite selection.

With regard to resin composite use, the most frequently reported factors influencing material selection were aesthetic expectations (77.7%) and durability (76.0%). Other factors included tooth position (63.0%) and follow-up duration/failure rate (34.3%), whereas patient age (12.9%) and cost (16.1%) were less frequently reported (Figure 3).

A total of 94.1% of participants reported that restorative material preferences varied according to cavity class, whereas 5.9% indicated no variation based on cavity classification.

In vital pulp therapy, the most frequently reported materials were calcium silicate–based materials (65.9%), followed by calcium hydroxide–based materials (51.9%) and glass ionomer cements (35.6%).

For patients with poor oral hygiene, amalgam (54.0%), glass ionomer cement (50.4%), and resin composite (42.7%) were the most commonly reported materials. Resin-modified glass ionomer (16.3%) and compomer (2.1%) were reported less frequently (Table 4).

An ordinal logistic regression model was initially tested. Although the model was found to be statistically significant (χ^2^(11) = 106.392, *p* < 0.001), the Test of Parallel Lines indicated that the proportional odds assumption was violated (χ^2^(33) = 67.554, *p* < 0.001). Therefore, multinomial logistic regression was applied to analyze knowledge level.

The multinomial model was statistically significant overall (χ^2^(10) = 89.051, *p* < 0.001). The pseudo R^2^ values were 0.235 for Cox & Snell, 0.268 for Nagelkerke, and 0.128 for McFadden.

According to the likelihood ratio tests, professional experience (χ^2^ = 37.835, *p* < 0.001), professional status (χ^2^ = 53.041, *p* < 0.001), and type of institution (χ^2^ = 6.320, *p* = 0.042) were identified as significant predictors of knowledge level.

Based on the parameter estimates, general dental practitioners were significantly less likely to have a high level of knowledge compared to specialists (OR ≈ 0.29, *p* < 0.001). No statistically significant difference was observed between research assistants and specialists in terms of achieving a high level of knowledge (*p* > 0.05).

Regarding professional experience, differences in the likelihood of achieving a high level of knowledge were observed across experience groups compared to the reference group. In particular, dentists with 16–20 years of experience showed a significantly higher likelihood of having a high level of knowledge (*p* < 0.05).

Although the type of institution was found to be significant in the overall model, it did not demonstrate consistent or clinically meaningful differences across pairwise comparisons of knowledge levels.

## 4. Discussion

In this study, the knowledge levels and clinical approaches of dentists actively practicing in Türkiye regarding evidence-based restorative material selection were evaluated. The findings indicate that although a considerable proportion of participants reported being familiar with the concept of evidence-based dentistry, their knowledge levels were predominantly self-rated as low to moderate.

Furthermore, the significant association between professional status and knowledge level, along with the observation of higher knowledge levels in certain experience groups, suggests that clinical experience and professional maturity may play a decisive role in evidence-based decision-making processes.

A study conducted by Lazarowitz et al. highlighted that the adoption of evidence-based practices among pediatric rehabilitation professionals remains limited, with considerable variability in knowledge, skills, and implementation levels among practitioners [16]. Similarly, as in other healthcare disciplines, these findings support the need to evaluate and strengthen the implementation of evidence-based practices in dentistry.

Clinical decision-making processes in restorative dentistry are often not based on strong scientific evidence, and considerable variability exists among clinicians in treatment approaches. Therefore, the evaluation and integration of evidence-based approaches are considered critical [17]. In a multi-country study conducted by Hatipoğlu et al. across 21 countries, restorative decision-making processes were shown to be influenced by material type, patient-related factors, and clinical experience. The authors also reported considerable heterogeneity between countries and emphasized the need for evidence-based clinical guidelines [13].

Similarly, a survey study conducted in Germany reported that dentists’ restorative material preferences vary depending on the clinical situation and may be influenced by practitioner-related factors [18]. Rath et al. demonstrated that, although evidence-based practices are recognized in dental education, their implementation in clinical practice remains limited [19].

In the present study, the finding that knowledge levels were predominantly low to moderate, along with the prominence of individual and practical factors in material selection, suggests that similar barriers to evidence-based practice may also exist in the Turkish context. This further indicates the absence of a universal standard in restorative material selection and highlights the need for evidence-based clinical guidelines.

In a large-scale study conducted in Jordan, Al-Asmar et al. reported a substantial gap between daily clinical practice and evidence-based approaches in restorative dentistry, with clinical decisions being influenced by both clinician experience and institutional practices [12].

Moreover, in a multinational study conducted within the framework of the European Regional Organization (ERO) of the World Dental Federation (FDI), Yamalik et al. reported that only approximately one-third of dentists were able to correctly define the concept of evidence-based dentistry, and only a limited proportion of participants were able to incorporate this approach into their daily clinical practice [9]. In addition, the indications for the use of restorative materials in anterior and posterior regions have been clearly outlined in clinical guidelines, taking into account cavity type, the amount of remaining tooth structure, and occlusal–functional risk factors [20].

Similarly, although a substantial proportion of participants in the present study reported being familiar with the concept of evidence-based dentistry, their knowledge levels were predominantly rated as low to moderate. In addition, the frequent reliance on peer opinions, social media content, short-term courses, and institutional habits in restorative material selection suggests that clinical decision-making in daily practice continues to be largely driven by individual experience rather than high-level evidence such as clinical guidelines, systematic reviews, and long-term survival data.

The preference for peer opinions and social media sources may be associated with practical factors such as heavy clinical workload, time constraints, and the ease of access to these resources. However, the lack of support from evidence-based guidelines may hinder the standardization of clinical decision-making.

This finding suggests that the evidence–practice gap, as reported in the international literature, also persists in the Turkish context. Therefore, it highlights the need to enhance the visibility of evidence-based guidelines and to strengthen structured continuing professional development programs based on these guidelines, in order to ensure that restorative material selection is aligned with biomechanical principles.

Staxrud et al. reported that, following clinical evaluation of cases requiring the replacement of existing restorations, tooth-colored direct restorations (predominantly composite) were recommended in approximately 74% of cases, whereas indirect restorations such as crowns were suggested in roughly one-quarter of cases [21]. In a cross-sectional study conducted in Saudi Arabia, Alsughair et al. reported that resin composites were the most commonly preferred materials for the restoration of Class I, II, and V primary carious lesions, with the primary rationale being the ability to perform conservative cavity preparations [22].

This finding, when considered alongside the present study—where aesthetic considerations and ease of use were prominent in material selection and composite materials were widely preferred—supports a global trend toward tooth-colored restorative materials in contemporary restorative dentistry.

In the present study, 94.1% of participants reported that their restorative material preferences varied according to cavity class. Resin-based materials were commonly preferred due to aesthetic expectations and durability. In contrast, the tendency to select amalgam and glass ionomer cement in patients with poor oral hygiene suggests that the use of more traditional materials persists under certain clinical conditions.

In recent years, global initiatives aimed at reducing the use of dental amalgam, particularly following the Minamata Convention, have gained increasing importance [14]. However, in Türkiye, amalgam is still included in undergraduate dental education and continues to be used in clinical practice [23]. The relatively high preference for amalgam among patients with poor oral hygiene is noteworthy. This finding suggests that, despite global policy changes, amalgam has not been completely abandoned in clinical practice and that dentists may adopt pragmatic approaches in material selection.

The continued preference for amalgam may be explained by its clinical advantages over resin composites, including greater durability, higher moisture tolerance, and lower technique sensitivity, particularly in patients with a high caries risk [24]. Furthermore, the preference for alternatives such as amalgam and glass ionomer cement over aesthetic materials in this patient group indicates that dentists consider not only aesthetic expectations but also caries risk, patient compliance, and the long-term success of the restoration.

A similar pattern can also be observed in material selection for vital pulp therapy. The present results demonstrate that calcium hydroxide–based materials continue to be preferred at a high rate. Although current literature indicates that calcium silicate–based materials (such as MTA and Biodentine) are superior in terms of biocompatibility, dentin bridge formation, and long-term clinical success [25,26], calcium hydroxide has not been completely abandoned in clinical practice.

This suggests that dentists consider not only scientific evidence but also their clinical experience, established habits, cost-related factors, and the ease of material handling when selecting materials. In particular, the long-standing use of calcium hydroxide, its wide availability, and its low cost may explain its continued preference.

However, considering the disadvantages of calcium hydroxide—such as its high solubility, reduced sealing ability over time, and its association with tunnel defects in dentin bridge formation—this material does not fully align with current evidence-based approaches [27]. Overall, these observations indicate that material selection in vital pulp therapy may still be influenced by traditional approaches and clinical habits rather than contemporary scientific evidence.

The literature indicates that the long-term success of restorations depends not only on the material used but also on patient- and tooth-related factors, as well as, importantly, the clinician’s decision-making processes [28]. Moreover, long-term practice-based studies have shown that the level of professional education and the adoption of up-to-date clinical approaches play a decisive role in improving the survival of restorations [29].

Consistent with these findings, the present study also showed that a substantial proportion of dentists rated their knowledge of evidence-based restorative material selection as low to moderate. The significant association between knowledge level and clinician-related variables, such as professional status and years of experience, suggests that clinical decision-making processes may play a potentially decisive role in restorative outcomes.

Moreover, the results of the multinomial regression analysis provide a more detailed understanding of these relationships. Although “type of institution” was identified as a statistically significant variable, its overall effect appears to be limited. This may be explained by the relatively homogeneous nature of undergraduate dental education in Türkiye, as well as shared clinical practices and similar continuing professional development opportunities across different institutions. In this context, the widespread use of common information sources—such as peer communication, professional courses, and digital platforms—may contribute to the convergence of knowledge levels among dentists, thereby reducing the practical impact of institutional differences.

The inadequacy of an evidence-based approach has significant implications not only for clinical practice but also at the level of the healthcare system and patient outcomes [30]. Frequent replacement of restorations may result in prolonged treatment times, increased costs, and a worsening of inequalities in access to care, particularly among individuals with high caries risk or those from disadvantaged socioeconomic backgrounds [28,31]. In addition, non-standardized decision-making processes and overly interventionist approaches among clinicians may lead to the unnecessary replacement of restorations and undermine patients’ trust in dental care [32,33].

Contemporary restorative dentistry is increasingly shaped by digital workflows. Intraoral scanners and CAD/CAM-based systems (e.g., 3Shape) enable three-dimensional evaluation of tooth structure, occlusion, and aesthetic parameters, thereby contributing to more predictable and standardized material selection. Similarly, digital smile design tools may support the integration of aesthetic expectations into treatment planning and influence the preference for tooth-colored restorative materials [34,35].

Although digital workflows were not directly evaluated in this study, the findings highlight the diversity of information sources involved in clinical decision-making. Within this context, digital technologies are expected to play an increasingly important role as supportive tools for evidence-based decision-making and may contribute to shaping restorative material preferences.

The present study provides a novel contribution to the literature by simultaneously evaluating evidence-based knowledge levels related to restorative material selection alongside material preferences, information sources, and demographic/professional variables within a single model, and by quantitatively highlighting the need for national guidelines in the Turkish context.

Within this framework, the findings indicate that evidence-based approaches to restorative material selection are not yet adequately reflected in clinical practice. The results of this survey may contribute to the restructuring of undergraduate curricula and continuing professional development programs in a way that strengthens evidence-based decision-making skills. Furthermore, the findings support the need for the development of nationally applicable, evidence-based restorative treatment guidelines to enhance consistency in clinical practice among practitioners. In this respect, the study serves as a data-driven reference for initiatives aimed at standardizing clinical decision-making processes.

## 5. Limitations

This study has several limitations that should be acknowledged. First, the cross-sectional design precludes any inference of causality. Second, the reliance on self-reported data introduces the potential for response and recall bias. In addition, as all variables were assessed using a single self-administered questionnaire completed in one session, the possibility of common method bias should be considered. Furthermore, self-reported knowledge levels may not fully reflect actual evidence-based competence, and this potential discrepancy should be taken into account when interpreting the findings.

Third, as the majority of participants were employed in public institutions, the findings may not be fully applicable to private or academic settings. It should also be noted that the relatively young age distribution of the sample, with nearly half of the participants having less than five years of professional experience, may limit the representativeness of the findings for more experienced clinicians.

Moreover, the questionnaire used in this study was not a previously validated standardized instrument but was developed specifically for the purposes of this research. Consequently, its psychometric properties, including internal consistency and construct validity, were not comprehensively assessed. However, the questionnaire was refined through pilot testing and expert review to ensure clarity and clinical relevance.

Given that knowledge of evidence-based practice and material preferences may vary across different clinical scenarios, caution is warranted when generalizing the findings to all restorative applications. Finally, because the proportional odds assumption was violated in the initially tested ordinal logistic regression model, multinomial logistic regression was adopted as the final analytical approach, and all interpretations are based on this model.

## 6. Conclusions

The findings of the present study suggest that restorative material selection in Türkiye is largely influenced by clinical experience, individual preferences, and habitual practices, whereas the integration of evidence-based decision-making may remain suboptimal. The observed association between knowledge levels and clinician-related variables, such as professional status and specific experience ranges, highlights the potential role of clinical maturity in shaping decision-making processes.

Material preferences appeared to vary according to key clinical factors, including aesthetic expectations, durability, and cavity characteristics, with a tendency toward more traditional materials in high-risk clinical conditions. Taken together, these findings highlight the need for the development of nationally applicable clinical guidelines and the implementation of structured educational programs—particularly targeting general dental practitioners—to improve consistency in clinical practice and support the integration of evidence-based approaches.

## Figures and Tables

**Table 1:** Demographic and professional characteristics of the study participants (*n* = 341).

Variable	*n*	%
Gender		
Female	142	41.6
Male	199	58.4
Age group (years)		
≤25	37	10.9
26–30	143	41.9
31–35	50	14.7
36–40	50	14.7
41–50	35	10.2
≥51	26	7.6
Professional experience (years)		
0–5	168	49.3
6–10	53	15.5
11–15	47	13.8
16–20	23	6.7
≥21	50	14.7
Type of institution		
Public hospital/Oral and Dental Health Center	174	51.0
University	91	26.7
Private practice	68	19.9
Other	8	2.3
Professional status		
General dental practitioner	233	68.3
Research assistant	53	15.5
Specialist	55	16.1

**Table 2:** Participants’ awareness of evidence-based dentistry (EBD) and self-reported knowledge levels (*n* = 341).

Variable	*n*	%
Awareness of EBD		
Yes	217	63.6
No	124	36.4
Total	341	100
Self-reported knowledge level of EBD		
Low (1–2)	156	45.8
Moderate (3)	121	35.4
High (4–5)	64	18.8
Total	341	100

Note: Frequencies (*n*) were derived from rounded percentages; the total sample size was 341.

**Table 3:** Sources from which participants acquired their knowledge of evidence-based dentistry (EBD) (*n* = 341).

Source of Knowledge	*n*	%
Undergraduate education—theoretical courses	203	59.5
Undergraduate education—clinical training	192	56.2
Clinical experience and peer opinions	198	58.0
Continuing professional courses/certification programs	118	34.6
Scientific journal articles	115	33.7
Professional meetings and congresses	110	32.2
Online educational platforms and webinars	42	12.4

Note: Percentages may exceed 100% because multiple responses were permitted. Frequencies (*n*) were derived from percentages based on the total sample size (*n* = 341) and rounded accordingly.

**Table 4:** Preferred restorative materials in vital pulp therapy and in patients with poor oral hygiene (*n* = 341).

Preferred Material	*n*	%
Materials Used in Vital Pulp Therapy (VPT)		
Calcium silicate–based materials (MTA, Biodentine, TheraCal)	225	65.9
Calcium hydroxide–based materials	177	51.9
Glass ionomer cement	121	35.6
Materials preferred in patients with poor oral hygiene		
Amalgam	184	54.0
Glass ionomer cement	172	50.4
Resin composite	146	42.7
Resin-modified glass ionomer	56	16.3
Compomer	7	2.1

Abbreviations: VPT, vital pulp therapy; MTA, mineral trioxide aggregate.

## Data Availability

The data presented in this study are available from the corresponding author upon reasonable request.
